# Safety of posterolateral approach with a polished cemented stem and a modular dual mobility implant in patients over 75 years old

**DOI:** 10.1016/j.jor.2026.02.042

**Published:** 2026-02-10

**Authors:** Cesare Meschini, Mattia Chirico, Matteo Innocenti, Giovanni Valentini, Paolo Salari, Andrea Baldini

**Affiliations:** a University of Florence, School of Human Health Sciences, Largo Brambilla 3, Florence 50134 Italy; b Department of Orthopedics and Geriatric Sciences, Catholic University of the Sacred Heart, Rome, Italy; c Department of Clinical Orthopaedics, AOU Careggi, University Hospiptal of Florence, Florence, Italy; d Istituto Fiorentino di Cura e Assistenza, 50139 Florence, Italy

**Keywords:** Dual mobility, Total hip arthroplasty, Cemented stem, modular dual mobility cup, Posterolateral approach, Elderly, Instability

## Abstract

**Background:**

With increasing life expectancy, total hip arthroplasty (THA) is increasingly performed in patients over 75 years, a population at higher risk of perioperative complications. This study assessed the safety and short-to mid-term outcomes of a standardized THA strategy in elderly patients using a posterolateral approach, a polished cemented stem, and—when indicated—a modular dual mobility (DM) cup.

**Methods:**

This retrospective multicenter study included patients >75 years who underwent primary THA with a cemented polished femoral stem and posterolateral approach (2017–2023). Group A received a modular DM cup, while Group B received a fixed-liner acetabular component. Clinical outcomes (Oxford Hip Score, VAS satisfaction) and complications were recorded. Survivorship was evaluated using Kaplan–Meier analysis in best- and worst-case scenarios.

**Results:**

642 THAs were analyzed (Group A: 460; Group B: 182). Groups were comparable in age and sex distribution. Dislocation rate was significantly lower with DM (0.7% vs 2.75%; p = 0.045). No differences were observed in infection, aseptic loosening, periprosthetic fracture, reoperation, readmission, or mortality. Postoperative OHS was similar, while patient satisfaction (VAS) was higher in Group A (93.0 ± 7.0 vs 81.9 ± 14.2; p < 0.001). Kaplan–Meier analysis demonstrated excellent survivorship in both cohorts, without significant differences. No intraprosthetic dislocations or DM-related mechanical failures occurred.

**Conclusion:**

THA using a posterolateral approach and a polished cemented stem is safe in patients over 75 years. Modular DM cups reduced dislocations and improved patient satisfaction without increasing complications. Their selective use may be advantageous in elderly, high-risk patients. Further prospective long-term studies are warranted.

**Level of evidence:**

Level III, retrospective cohort study.

## 1 Introduction

Advancements in healthcare have significantly increased life expectancy, leading to a rise in elective arthroplasty procedures, such as hip and knee replacements, being performed on individuals well into their ninth decade of life 1, 2, 3, 4, 5. As the aging population grows, the number of patients over the age of 75 seeking total hip arthroplasty (THA) has also increased. This trend raises important concerns regarding the safety and feasibility of major orthopedic surgeries, like total joint replacements, in this older demographic. This age group is associated with a higher risk of perioperative complications within the first 30 days.^6^

Several modifiable peri-operative parameters may be targeted to mitigate these issues. Three primary intra-operative factors are relevant to the implant procedure: the type of approach, the type of fixation and the type of articulation.

Several surgical approaches are described for THA procedures. Among these, the anterior approach has gained popularity in recent years due to its association with faster functional recovery and a lower risk of dislocation. However, it is also linked to a higher risk of periprosthetic fractures and has a steeper learning curve 7, 8, 9. The anterolateral and direct lateral approaches similarly offer a reduced risk of dislocation but are associated with a risk of damage to the abductor mechanism 10, 11, 12. The posterolateral approach, despite having the highest dislocation risk among these options, provides superior surgical exposure, effective hemostasis, is generally performed rapidly, and is easily extendable 13, 14, 15.

The optimal type of fixation remains a subject of ongoing debate, particularly in the elderly population. Several studies have indicated that cemented femoral stems are associated with a lower perioperative periprosthetic fracture rate compared to cementless stems.^16^^,^^17^ However, cementless stems offer advantages in terms of osseointegration and superior long-term durability. Additionally, cemented stems provide immediate implant stability and are linked to reduced thigh pain and a reduce incidence of stress shielding.^18^^,^^19^

In terms of articulation type, a primary distinction can be made between fixed liner and dual-mobility designs. The fixed liner offers several advantages, including the ability to use screws for enhanced fixation, the capability to verify the proper positioning of the component, the possibility of isolated insert revision, and the ability to accommodate diverse insert materials for the insert. However, it is associated with a higher risk of dislocation compared to dual-mobility designs. Dual-mobility components are available in both monoblock and modular configurations. Modular dual-mobility implants offer similar benefits to the fixed liner, including the ability to revise components, greater elasticity than the monobloc design.^20^ Potential issues with modular dual-mobility components include the potential metal ions release, fretting, liner malposition potentially resulting in dislocation, and intraprosthetic dislocation, and reduced anti-luxation properties compared to the monoblock counterparts.^21^, ^22^, ^23^, ^24^

The aim of this study is to assess whether the combination of the postero-lateral approach with a polished cemented femoral stem in THA constitutes a safe procedure in the population over 75 years of age, and if the routinary addition of a modular dual-mobility cup could improve the results further.

## 2 Materials and methods

This study employed a retrospective, multicenter observational design, utilizing patient data collected from two hospitals’ orthopedic departments (Casa di cura Ulivella e Glicini – Firenze and Azienda Ospedaliero Universitaria Careggi – Firenze) between January 2017 and December 2023. The study adhered to the ethical principles established in the Declaration of Helsinki and received approval from the local Ethics Committee (approval code 27764_oss).

To meet the study's goals, we conducted a systematic search of institutional registries. This systematic database review allowed us to collect and record detailed patient data. This data included detailed demographic data, such as age, gender and side of planned surgery. The inclusion criteria comprised individuals with age >75 years, who underwent THA for primary or secondary hip arthrosis, for which a cemented femoral stem was used and at least 1 year of follow-up. Conversely, exclusion criteria encompassed cases with incomplete data, age <75 years, use of uncemented femoral stem, different approach than posterolateral, less than one year of follow-up.

In group A, all patients received a titanium alloy 3D-printed porous dual-mobility acetabular cup (G7 acetabular system; ZimmerBiomet Inc., Warsaw, IN, USA), combined with a 28-mm head for acetabular components larger than 48 mm or a 22-mm head for smaller components and a cemented straight polished femoral stem component (Versys Heritage - ZimmerBiomet Inc., Warsaw, IN, USA).

In group B, all patients received a cup with fixed liner (G7 acetabular system - ZimmerBiomet Inc., Warsaw, IN, USA) and a cemented straight polished femoral stem component (VersysHeritage - ZimmerBiomet Inc., Warsaw, IN, USA) combined with a ceramic head (28 mm in 21 cases - 11,5%, 32 mm in 109 cases - 59.9%, and 36 mm in 52 cases - 28,5%) and a polyethylene liner (10° elevated rim in 161 cases - 88,5%, neutral in 21 cases - 11,5%).

In both groups all cases were performed by experienced orthopedic surgeons in hip arthroplasty. A standard posterolateral approach was used in all cases. To prevent infection, all patients received a preoperative dose of cefazolin unless there were allergies or other medical reasons to avoid it. On the day of surgery, patients were allowed to walk fully weight-bearing with the support of two crutches. All patients followed the same standardized fast track rehabilitation protocol. Clinical evaluations were conducted in an outpatient setting at 20, 45 days, 6 months and yearly thereafter.

We employed the Oxford Hip Score (OHS)^25^ to evaluate patient-reported outcomes before and after surgical intervention and the Visual Analogue Scale (VAS) for satisfaction^26^ and a 5-points Likert scale (0 = very dissatisfied, 1 = dissatisfied, 2 = neutral, 3 = satisfied, 4 = very satisfied) to assess patient satisfaction.

Any complications including dislocation, infection, periprosthetic fracture, aseptic mobilization, wound dehiscence, re-hospitalization within 30 days, subjective heterometry greater than 1 cm and the need for re-intervention were recorded for each patient.

### 2.1 Statistical analysis

IBM SPSS statistics software (Version 26.0, IBM) was used to perform the statistical analysis. Based on different distributions of outcome variables, the Student's t-test and Mann–Whitney *U* test were used for comparisons of clinical outcomes between the two groups. The χ2 test was used for categorical data between groups, and Fisher's exact test was applied when the expected frequencies were too low for the χ^2^ test to be reliable. Individual characteristics, including age, BMI, sex, side operated were considered for descriptive analysis. A p value of <0.05 was considered statistically significant.

A per-protocol analysis was conducted, including only participants with available follow-up data. A comparison between participants lost to follow-up and those retained revealed no significant differences in baseline sociodemographic and clinical variables; however, a significant difference in loss to follow-up rates was observed between the two centers. This suggests that the missing data may not be Missing Completely At Random (MCAR), but rather Missing At Random (MAR), warranting caution in the interpretation of results and consideration of the study center in subsequent analyses.

Based on the overall reintervention rate (8 out of 642; 1.25%), an estimated 0.3 out of 26 participants lost to follow-up would be expected to have undergone reintervention, assuming the same rate applies. This suggests that the impact of loss to follow-up on the reintervention outcome is likely negligible.

## 3 Results

A total of 460 patients were included in Group A and 182 in Group B. The mean age was comparable between the two groups (80.12 ± 4.07 vs. 79.81 ± 3.96 years; *p* = 0.389), as was sex distribution (female: 74.1% vs. 68.7%; *p* = 0.170) and operated side (left: 45.9% vs. 43.4%; *p* = 0.598). Group A had a significantly higher BMI (27.14 ± 6.11 vs. 25.92 ± 4.73; *p* = 0.016), whereas a significantly greater proportion of patients in Group B were classified as ASA 3 (11.3% vs. 45.6%; *p* < 0.001).

A significant difference was observed between groups in terms of dislocation (0.7% vs. 2.75%; p = 0.045). No significant differences were observed between the groups in terms of periprosthetic joint infection (0.2% vs. 0%; *p* = 1), aseptic loosening (0.2% vs. 0%; *p* = 1), periprosthetic fracture (0.2% vs. 1.1%; *p* = 0.206), reoperation rates (1.1% vs. 1.7%; *p* = 0.068), wound dehiscence (0.9% vs. 1.1%; *p* = 1), heterometry >1 cm (1.4% vs. 0.6%; *p* = 1), 30-day readmission (0.9% vs. 0.5%; *p* = 0.057), or mortality (7.1% vs. 5.6%; *p* = 0.595). However, Group A had a significantly higher rate of patients lost to follow-up (5.2% vs. 1.1%; *p* = 0.014).

Regarding 30-day rehospitalizations, in group A these included atrial fibrillation (n = 1), ureteral obstruction from renal calculi (n = 1), a non-periprosthetic pelvic fracture following a fall (n = 1), and incidental detection of a pancreatic cyst (n = 1). In group B, one patient was rehospitalized for hematoma drainage related to the surgical procedure.

The mean follow-up duration was longer in Group A (41.63 ± 20.88 vs. 35.71 ± 14.43 months; *p* = 0.001), as was the follow-up until any event occurred (41.17 ± 20.89 vs. 35.43 ± 14.6 months; *p* = 0.001). Preoperative OHS were significantly better in Group A (23.12 ± 3.4 vs. 20.53 ± 1.9; *p* < 0.001), while postoperative scores were comparable between groups (42.14 ± 3.1 vs. 41.92 ± 1.8; *p* = 0.368). Patient-reported satisfaction was significantly higher in Group A (*p* < 0.001), with 98.8% reporting a satisfaction score ≥3 compared to 93.9% in Group B. VAS scores were also significantly better in Group A (93.05 ± 7.01 vs. 81.94 ± 14.2; *p* < 0.001).

All data are presented in Table 1.

**Table 1:**
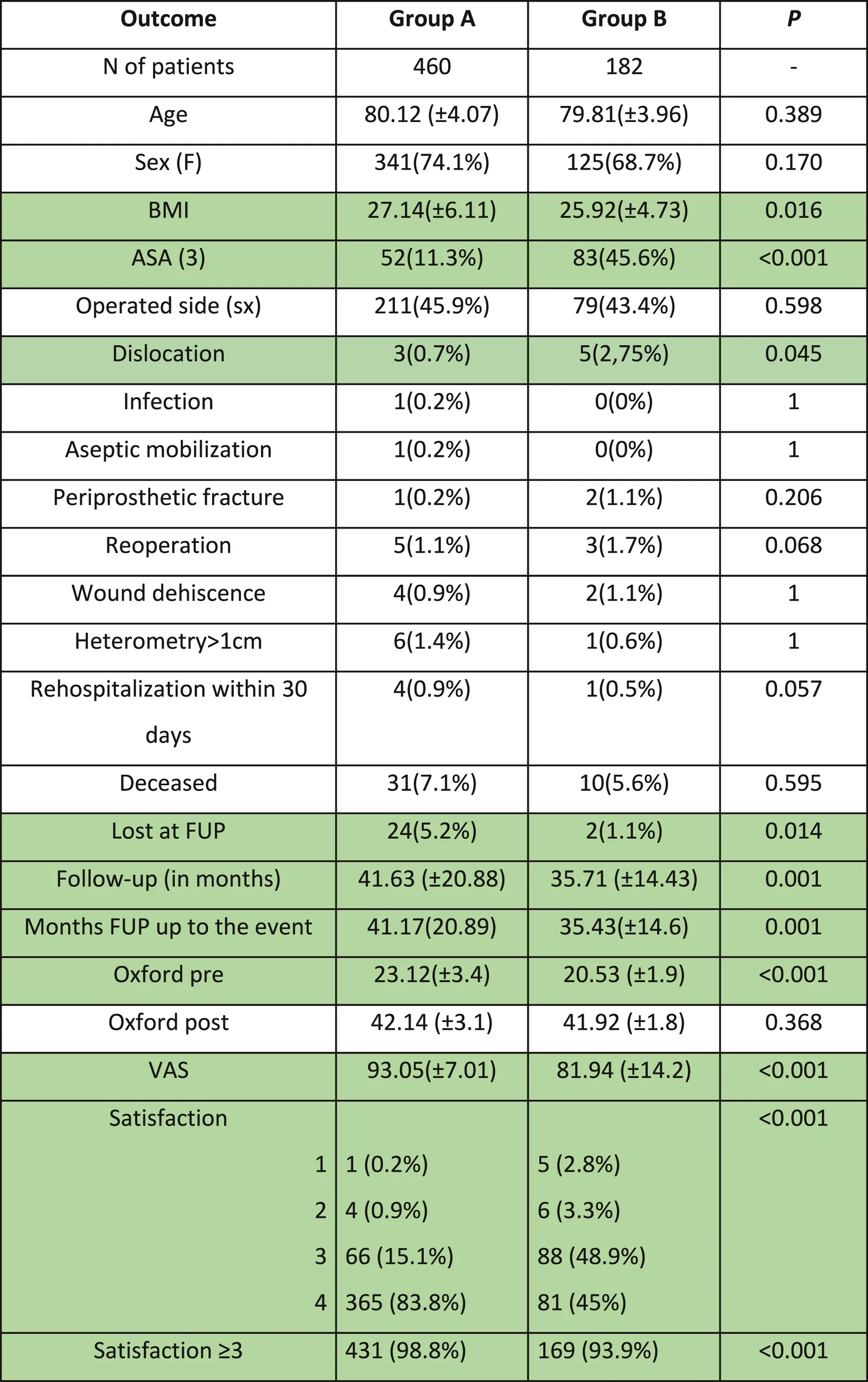
Comparison between the two orthopedic institutes. Dichotomous variables were reported as frequency and percentage; Age, BMI, Follow-up (in months) Oxford-pre and post, and VAS were reported as mean (± standard deviation).

In the present study, two Kaplan–Meier survival curves were generated to compare outcomes between Group A and Group B.

The first curve represents the best-case scenario, in which only reinterventions were considered as events. All patients from both cohorts were included in the analysis. Censored cases included deaths, losses to follow-up, and patients without events at the final follow-up.

The second curve corresponds to the worst-case scenario, in which both reinterventions and deaths were considered as failures (events). Additionally, patients lost to follow-up were assigned an estimated failure rate equivalent to that of the overall cohort, in line with the assumption of data missing at random (MAR).

Both curves were used to assess and compare long-term survivorship of the implants in the two groups.

**Fig. 1:**
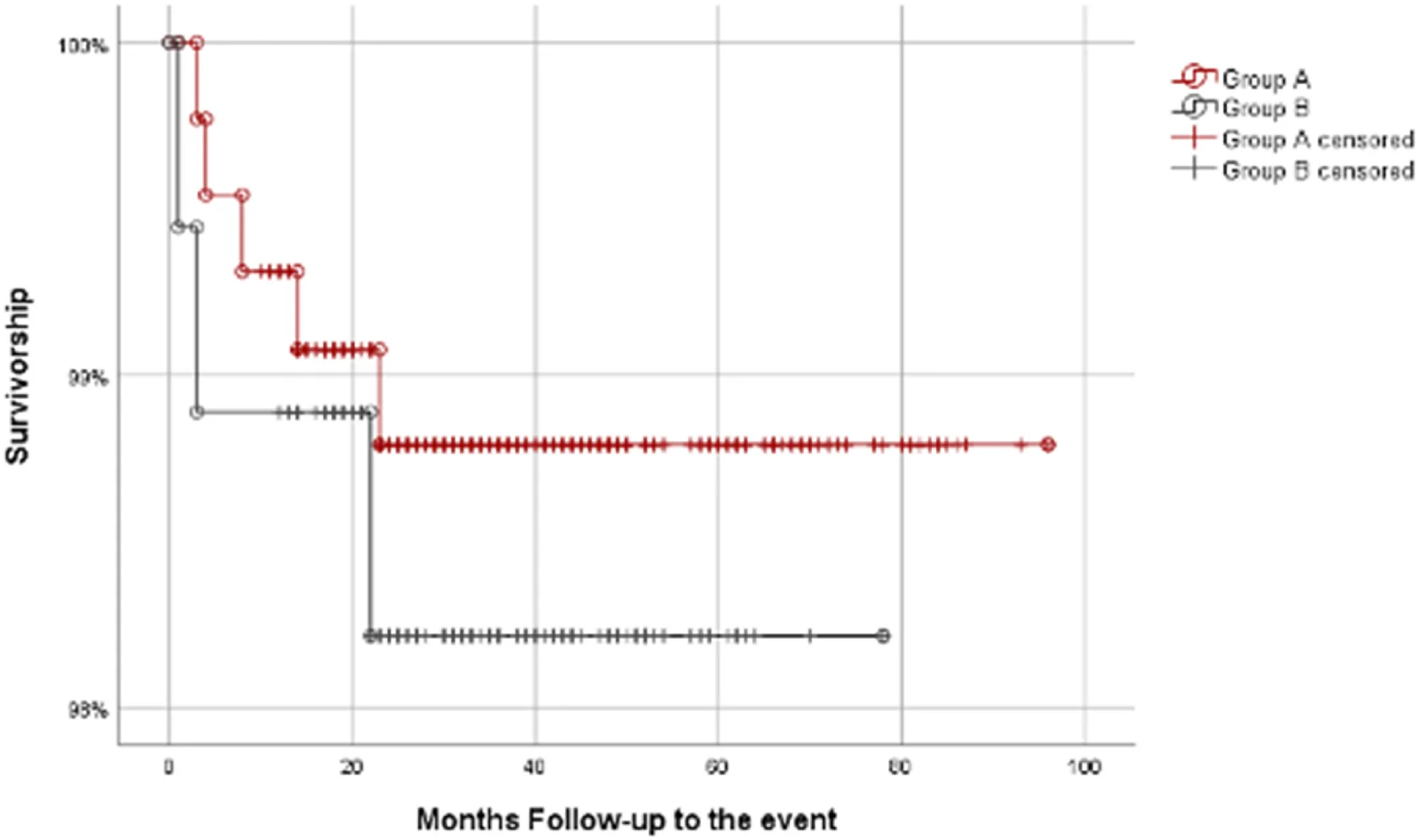
Survivorship comparison between two institutes in best scenario (figure enlarged along the y-axis to highlight minimal variations in survival between 98% and 100%.) Group A estimated survival rate: 98.8% - mean estimated survival rates 94.97% [CI95: 95.87-90.08] Group B estimated survival rate: 98.2% mean estimated survival rates 76.78% [CI95: 78.15-75.41] p = 0.605.

In the best-case scenario Kaplan-Meier survival analysis (Fig. 1), Group A demonstrated excellent survivorship at all time points. At 1 year, the estimated survival rate was 99.8% for Group A and 99.4% for Group B (p = 0.518). At 3 years, survival remained high in both cohorts, with estimates of 99.5% for Group A and 98.8% for Group B (p = 0.362). At 5 years, the estimated survival rate was 99.5% for Group A and 95.7% for Group B, with a trend toward significance (p = 0.102). Although differences did not reach statistical significance, Group A consistently showed higher estimated survival rates over time compared to Group B.

**Fig. 2:**
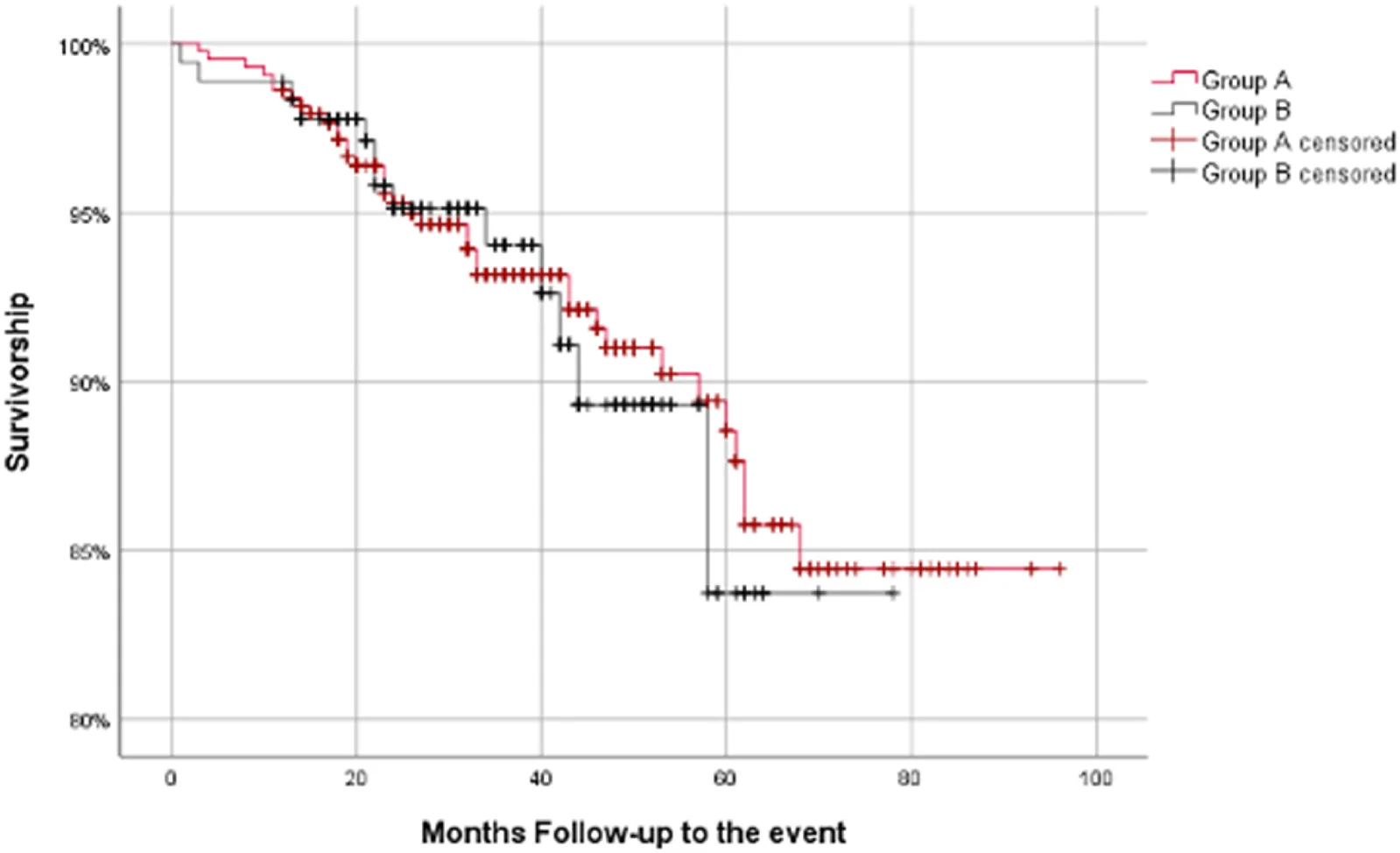
Survivorship between two institutes in worst scenario Group A estimated survival rate: 78.1% - mean estimated survival rates 87.44% [CI95: 90.13-84.75] Group B estimated survival rate: 83.7% mean estimated survival rates 71.7% [CI95: 75.2-68.16] p = 0.847.

In the worst-case scenario Kaplan-Meier survival analysis (Fig. 2), Group A exhibited a estimated survival rate of 99.1% at 1 year, compared to 99.4% in Group B (p = 0.651). At 3 years, the survival rate was 93.9% for Group A and 94.6% for Group B (p = 0.773). At 5 years, Group A showed a estimated survival rate of 89.3%, while Group B demonstrated a lower survival of 81.7% (p = 0.499). In our series, Kaplan–Meier analysis confirmed excellent mid-term survivorship of the DM construct, with significantly higher estimated survival rates compared to standard liners in both best- and worst-case scenarios. These results support the mechanical integrity and biocompatibility of modular DM designs, with no evidence of increased wear, metal ion release, or intraprosthetic dislocation, in agreement with prior reports.

## 4 Discussion

This study evaluated the safety and short-to mid-term clinical performance of a standardized total hip arthroplasty (THA) protocol in patients aged over 75 years, combining a posterolateral approach with a polished cemented femoral stem and a modular dual mobility (DM) acetabular component. The comparison between two cohorts—one receiving a DM cup (group A) and one receiving a standard cup (group B)—revealed superior outcomes in the DM group, confirming the hypothesis that dual mobility constructs may offer clinical benefit in elderly high-risk patients. Patients treated in group A, where the DM implant was used systematically, exhibited better postoperative pain control (VAS), higher satisfaction, and improved functional recovery compared to those receiving a fixed liner.

Dislocation remains one of the most frequent and disabling complications following THA, especially in older patients who often exhibit diminished neuromuscular control and sarcopenia. In the present study, the dislocation rate was 0.7% in the dual mobility (DM) group and 2.75% in the standard cup group (p = 0.045), confirming the superior stability of the DM construct in an elderly population.

This finding aligns with previous registry-based and institutional reports highlighting the benefit of DM designs in reducing instability. For example, Hoskins,^27^ analyzing AOANJRR data, found that while overall revision rates did not differ significantly between DM and large-head bearings, DM cups significantly reduced dislocation-related revisions in acetabular components <58 mm (0.3% for DM vs 0.6% for large-head bearings). Hussein 28 similarly demonstrated a lower risk of dislocation with DM cups compared to 36-mm heads in primary THA for osteoarthritis, without an increase in other complications. Pellegrini^29^ reported favorable short-to mid-term outcomes using a hybrid construct combining a cementless hemispherical DM cup with a cemented polished femoral stem in elderly patients, observing no intraprosthetic dislocations and very low rates of instability.

By contrast, in elderly patients treated with conventional implants through a posterior approach, the dislocation rate remains considerably higher. Hermansen^30^ reported a “true” 1-year incidence of 2.8% for all dislocations (including closed reductions) following primary THA, most of which were performed through a posterior approach, underscoring the intrinsic instability risk associated with this surgical route in frail or sarcopenic populations.

The dislocation rate observed in our series is consistent with these data and may reflect the characteristics of our cohort, consisting of octogenarian patients with lower compliance and diminished neuromuscular control. Importantly, the dual mobility construct appears to mitigate this risk effectively, likely due to its increased jump distance and secondary articulation, which enhance joint stability without compromising range of motion or implant fixation. Concerns regarding potential complications such as excessive wear or elevated serum metal ion levels have not been substantiated; both modular and monoblock DM systems show uniformly low ion levels comparable to standard implants 20, 21, 22, 23, 24.

Overall, our results reinforce the growing body of evidence supporting the use of DM cups in primary THA for elderly patients, particularly when the posterior approach is employed, where the risk of instability is intrinsically higher.

Smaller clinical studies have provided further evidence for the mechanical reliability of hybrid DM constructs. Prudhon^31^ reported a six-year implant survival rate of 98.4% in patients treated with a cemented stem and DM cup, with only isolated cases of deep infection, dislocation, and periprosthetic fracture. Similarly, Caton^32^ documented a ten-year dislocation rate of just 0.9% with DM cups used alongside a cemented Charnley-type stem, compared to 12.9% in a cohort treated with standard polyethylene bearings. In a comparative study by Ebied,^33^ none of the cemented DM femoral stems required revision, while one uncemented stem failed, and four intraoperative fractures occurred in the uncemented group, none of which compromised stability.

The use of cemented femoral fixation in elderly patients is supported by substantial registry-based evidence, which consistently shows higher revision rates for uncemented stems due to complications such as periprosthetic fractures and early loosening. Kelly,^34^ using data from the American Joint Replacement Registry, found that cementless stems were associated with a 7.7-fold increased risk of periprosthetic fracture in patients aged 65 and older. Advanced age and female sex were also identified as independent risk factors. A similar trend was observed by Brüggemann,^35^ who reported a higher incidence of intraoperative fractures with cementless fixation, particularly in patients over 80 years of age. These results are reinforced by the findings of Tanzer,^36^ who showed that cemented stems had a significantly lower early revision rate than cementless stems, particularly in the first three postoperative months. Babazadeh,^37^ in a large registry analysis from the AOANJRR, demonstrated that cemented femoral components consistently exhibited lower revision rates across all time intervals, with the most significant differences occurring in the immediate postoperative period.

In large registry-based studies, the reported incidence of femoral periprosthetic fractures in elderly patients ranges between 1.5% and 3%, and aseptic loosening between 0.5% and 1.2% 34, 35, 36, 37. In our series, the rate of femoral periprosthetic fracture was markedly lower—0.22% in the dual mobility cohort (1 case out of 460) and 0.47% when considering the entire population (3 cases out of 642, including one intraoperative event)—with no cases of aseptic loosening of the femoral stem observed during follow-up. These data confirm the excellent mechanical stability and safety of cemented stem fixation in this elderly cohort.

The mechanical advantages of cemented polished stems, particularly when implanted using the French Paradox technique,^38^ are well-documented. This method allows for immediate fixation and reduced micromotion, while simultaneously minimizing the risk of intraoperative and early postoperative fractures in osteoporotic bone. Furthermore, the consistent use of a posterolateral approach across all cases in this study likely contributed to the reproducibility of outcomes and may synergize well with dual mobility systems in reducing the risk of posterior construct instability.

Taken together, these data strongly favor the use of cemented fixation—particularly in patients over 75 or with osteoporotic bone—as a means to improve early mechanical stability and reduce the risk of revision surgery due to periprosthetic fracture or early loosening. In the overall series (642 cemented stems), only three femoral periprosthetic fractures (0.47%)—including one intraoperative event—and no cases of aseptic loosening were recorded, further supporting the safety and durability of cemented femoral fixation in elderly patients.

From an organizational and healthcare management standpoint, optimizing the surgical construct for elderly patients—through the systematic use of cemented femoral stems and dual mobility (DM) cups within a enhanced recovery after surgery (ERAS) model—may lead to reduced complication rates, improved functional recovery, and shorter hospital stays. These factors are particularly relevant in geriatric populations, who typically present with limited physiological reserves and are especially vulnerable to the adverse effects of prolonged hospitalization.

Several economic efficiency studies support this approach. In particular, the use of DM bearings has been shown to be economically advantageous, despite higher upfront implant costs, due to the reduction in dislocation rates and subsequent revision surgeries ^39^^,^^40^. This is especially true in high-risk populations or settings where instability-related complications carry substantial clinical and financial consequences ^41^^,^^42^.

Taken together, the integration of DM cups and cemented stems within an enhanced recovery framework appears not only clinically advantageous but also economically rationale, particularly when applied in a targeted elderly, high-risk population.

Strengths of this study include its focus on a high-risk population (>75 years of age), the consistent application of surgical and perioperative protocols across two high-volume centers, and a comparative design that reflects current clinical practice. These elements enhance the external validity and applicability of our findings.

However, several limitations should be acknowledged. The retrospective nature of the study introduces potential selection bias, and the absence of randomization precludes definitive causal inferences. Although long-term data are not yet available, the superior short-to mid-term outcomes observed with the DM construct strongly support its use in elderly patients. Additionally, long-term functional and radiographic data were not available, and although no cases of intraprosthetic dislocation or late mechanical failure were observed, the duration of follow-up period may be insufficient to capture late-onset complications. Moreover, operative procedures were performed by multiple surgeons from two different hospitals, which may have introduced variability in surgical technique and perioperative management despite adherence to standardized protocols.

Future research should focus on long-term implant survivorship, patient-reported functional outcomes, and economic efficiency analyses of DM constructs in geriatric THA. Randomized prospective trials comparing dual mobility to standard cups in high-risk populations are needed to inform evidence-based implant selection and optimize care pathways in this growing patient group.

## 5 Conclusion

In patients over 75 years of age undergoing total hip arthroplasty for primary osteoarthritis, the combination of a posterolateral approach, a polished cemented femoral stem, and a modular dual mobility acetabular component appears to be a safe and effective surgical strategy. Despite a more complex preoperative profile, patients receiving the dual mobility construct reported better postoperative pain control and higher satisfaction, with no increase in complication rates during follow-up. These findings support the selective use of dual mobility implants in elderly high-risk populations, although prospective studies with longer follow-up are needed to confirm long-term safety, functional benefits, and economic efficiency.

## Guardian/patient's consent

Informed Consent has been obtained from patient or guardian for the study's participation and publication.

## Author contributions

Conceptualization: C. Meschini, M. Chirico, A. Baldini; Methodology: G. Valentini, M. Chirico; Investigation: C. Meschini, M. Chirico, M. Innocenti, P. Salari; Data Curation: C. Meschini, M. Chirico; Formal Analysis: M. Chirico; Writing – Original Draft: C. Meschini, M. Chirico; Writing – Review & Editing: G. Valentini, A. Baldini; Supervision: G. Valentini.

## Ethics statement

This study, titled “Safety of Posterolateral approach with a polished cemented stem and a modular dual mobility implant in patients over 75 years old”, was conducted in accordance with the ethical standards of the institutional and national research committees and with the 1964 Helsinki Declaration and its later amendments.

Ethical approval was obtained from the local Ethics Committee Comitato Etico Regione Toscana N° 27764_oss. March 19, 2025.

All participants provided informed consent prior to inclusion in the study. Patient data were anonymized before analysis to ensure confidentiality. No additional interventions, beyond standard clinical practice, were performed for research purposes.

## Funding statement

This research did not receive any specific grant from funding agencies in the public, commercial or not-for-profit sectors.

## Conflict of interests

The authors declare that they have no known competing financial interests or personal relationships that could have appeared to influence the work reported in this paper.

## Abbreviations

**ASA**: American Society of Anesthesiologists
**BMI**: Body Mass Index
**CI**: Confidence Interval
**DM**: Dual Mobility
**ERAS**: Enhanced Recovery After Surgery
**FUP**: Follow-up
**MCAR**: Missing Completely At Random
**MAR**: Missing At Random
**OHS**: Oxford Hip Score
**PJI**: Periprosthetic Joint Infection
**THA**: Total Hip Arthroplasty
**VAS**: Visual Analogue Scale
